# Supplementary material for: Resolving the full spectrum of human genome variation using Linked-Reads
Source: Genome Res. 2019 Apr;29(4):635–45. doi: 10.1101/gr.234443.118 (PMC6442396; doi:10.1101/gr.234443.118)
Supplement: Supplemental Material [file supp_gr.234443.118_Supplemental_longranger-2.2.zip › longranger-2.2.tar.bz2!/lr2.2-public/lib/stan/cmdstan/stan/doc/api/html/agrad_2rev_2matrix_2log__determinant_8hpp_source.html]

Stan: src/stan/agrad/rev/matrix/log\_determinant.hpp Source File


|  |  |
| --- | --- |
|  | Stan  2.5.0  probability, sampling & optimization |


- Main Page
- Related Pages
- Namespaces
- Classes
- Files

- File List
- File Members

All Classes Namespaces Files Functions Variables Typedefs Enumerations Enumerator Friends Macros Pages

- src
- stan
- agrad
- rev
- matrix

log\_determinant.hpp

Go to the documentation of this file.

1 #ifndef STAN\_\_AGRAD\_\_REV\_\_MATRIX\_\_LOG\_DETERMINANT\_HPP

2 #define STAN\_\_AGRAD\_\_REV\_\_MATRIX\_\_LOG\_DETERMINANT\_HPP

3

4 #include <vector>

5 #include <stan/math/matrix/Eigen.hpp>

6 #include <stan/math/matrix/typedefs.hpp>

7 #include <stan/agrad/rev/var.hpp>

8 #include <stan/agrad/rev/matrix/typedefs.hpp>

9 #include <stan/math/error\_handling/matrix/check\_square.hpp>

10

11 // FIXME: use explicit files

12 #include <stan/agrad/rev.hpp>

13

14 namespace stan {

15  namespace agrad {

16

17  namespace {

18  template<int R,int C>

19  class log\_determinant\_vari : public vari {

20  int \_rows;

21  int \_cols;

22  double\* A\_;

23  vari\*\* \_adjARef;

24  public:

25  log\_determinant\_vari(const Eigen::Matrix<var,R,C> &A)

26  : vari(log\_determinant\_vari\_calc(A)),

27  \_rows(A.rows()),

28  \_cols(A.cols()),

29  A\_((double\*)stan::agrad::memalloc\_.alloc(sizeof(double)

30  \* A.rows() \* A.cols())),

31  \_adjARef((vari\*\*)stan::agrad::memalloc\_.alloc(sizeof(vari\*)

32  \* A.rows() \* A.cols()))

33  {

34  size\_t pos = 0;

35  for (size\_type j = 0; j < \_cols; j++) {

36  for (size\_type i = 0; i < \_rows; i++) {

37  A\_[pos] = A(i,j).val();

38  \_adjARef[pos++] = A(i,j).vi\_;

39  }

40  }

41  }

42  static

43  double log\_determinant\_vari\_calc(const Eigen::Matrix<var,R,C> &A)

44  {

45  Eigen::Matrix<double,R,C> Ad(A.rows(),A.cols());

46  for (size\_type j = 0; j < A.cols(); j++)

47  for (size\_type i = 0; i < A.rows(); i++)

48  Ad(i,j) = A(i,j).val();

49  return Ad.fullPivHouseholderQr().logAbsDeterminant();

50  }

51  virtual void chain() {

52  using Eigen::Matrix;

53  using Eigen::Map;

54  Matrix<double,R,C> adjA(\_rows,\_cols);

55  adjA = adj\_

56  \* Map<Matrix<double,R,C> >(A\_,\_rows,\_cols)

57  .inverse().transpose();

58  size\_t pos = 0;

59  for (size\_type j = 0; j < \_cols; j++) {

60  for (size\_type i = 0; i < \_rows; i++) {

61  \_adjARef[pos++]->adj\_ += adjA(i,j);

62  }

63  }

64  }

65  };

66  }

67

68  template <int R, int C>

69  inline var log\_determinant(const Eigen::Matrix<var,R,C>& m) {

70  stan::math::check\_square("log\_determinant(%1%)",m,"m",(double\*)0);

71  return var(new log\_determinant\_vari<R,C>(m));

72  }

73

74  }

75 }

76 #endif

typedefs.hpp

typedefs.hpp

\_adjARef

vari \*\* \_adjARef

**Definition:** log\_determinant.hpp:23

stan::agrad::inverse

Eigen::Matrix< fvar< T >, R, C > inverse(const Eigen::Matrix< fvar< T >, R, C > &m)

**Definition:** inverse.hpp:21

\_rows

int \_rows

**Definition:** log\_determinant.hpp:20

stan::agrad::memalloc\_

memory::stack\_alloc memalloc\_

**Definition:** var\_stack.cpp:16

rev.hpp

stan::agrad::size\_type

Eigen::Matrix< double, Eigen::Dynamic, Eigen::Dynamic >::Index size\_type

**Definition:** typedefs.hpp:14

stan::math::rows

size\_t rows(const Eigen::Matrix< T, R, C > &m)

**Definition:** rows.hpp:12

var.hpp

stan::math::cols

size\_t cols(const Eigen::Matrix< T, R, C > &m)

**Definition:** cols.hpp:12

Eigen.hpp

stan::agrad::var

Independent (input) and dependent (output) variables for gradients.

**Definition:** var.hpp:27

\_cols

int \_cols

**Definition:** log\_determinant.hpp:21

A\_

double \* A\_

**Definition:** log\_determinant.hpp:22

stan::agrad::log\_determinant

fvar< T > log\_determinant(const Eigen::Matrix< fvar< T >, R, C > &m)

**Definition:** log\_determinant.hpp:20

stan::math::check\_square

bool check\_square(const char \*function, const Eigen::Matrix< T\_y, Eigen::Dynamic, Eigen::Dynamic > &y, const char \*name, T\_result \*result)

Return true if the specified matrix is square.

**Definition:** check\_square.hpp:24

check\_square.hpp


---

[ Stan Home Page ]

*© 2011–2014,
Stan Development Team.*
